# Application of Multivariate Adaptive Regression Splines (MARSplines) for Predicting Antitumor Activity of Anthrapyrazole Derivatives

**DOI:** 10.3390/ijms23095132

**Published:** 2022-05-04

**Authors:** Marcin Gackowski, Karolina Szewczyk-Golec, Robert Pluskota, Marcin Koba, Katarzyna Mądra-Gackowska, Alina Woźniak

**Affiliations:** 1 Department of Toxicology and Bromatology, Faculty of Pharmacy, L. Rydygier Collegium Medicum in Bydgoszcz, Nicolaus Copernicus University in Torun, A. Jurasza 2 Street, PL-85089 Bydgoszcz, Poland; pluskota.r@gmail.com (R.P.); kobamar@cm.umk.pl (M.K.); 2 Department of Medical Biology and Biochemistry, Faculty of Medicine, L. Rydygier Collegium Medicum in Bydgoszcz, Nicolaus Copernicus University in Torun, Karłowicza 24 Street, PL-85092 Bydgoszcz, Poland; karosz@cm.umk.pl (K.S.-G.); al1103@cm.umk.pl (A.W.); 3 Department of Geriatrics, Faculty of Health Sciences, L. Rydygier Collegium Medicum in Bydgoszcz, Nicolaus Copernicus University in Torun, Skłodowskiej Curie 9 Street, PL-85094 Bydgoszcz, Poland; katarzyna.madra@cm.umk.pl

**Keywords:** anthrapyrazoles, antitumor activity, multivariate adaptive regressions splines, quantitative structure-activity relationships (QSAR)

## Abstract

An approach using multivariate adaptive regression splines (MARSplines) was applied for quantitative structure–activity relationship studies of the antitumor activity of anthrapyrazoles. At the first stage, the structures of anthrapyrazole derivatives were subjected to geometrical optimization by the AM1 method using the Polak–Ribiere algorithm. In the next step, a data set of 73 compounds was coded over 2500 calculated molecular descriptors. It was shown that fourteen independent variables appearing in the statistically significant MARS model (i.e., descriptors belonging to 3D-MoRSE, 2D autocorrelations, GETAWAY, burden eigenvalues and RDF descriptors), significantly affect the antitumor activity of anthrapyrazole compounds. The study confirmed the benefit of using a modern machine learning algorithm, since the high predictive power of the obtained model had proven to be useful for the prediction of antitumor activity against murine leukemia L1210. It could certainly be considered as a tool for predicting activity against other cancer cell lines.

## 1. Introduction

Anthrapyrazoles are synthetic anticancer drugs, synthesized in order to retain high levels of the wide spectrum of antitumor activity in anthracyclines (e.g., doxorubicin), while at the same time, diminishing cardiotoxicity by reducing the potential to generate semiquinone free radicals in cardiac cells [1,2]. Although there was a broad range of antitumor activity in model tumors [1,3], they revealed diversified activity in doxorubicin-resistant cells [4]. The action mechanism of these planar compounds is based on DNA intercalation, topoisomerase II inhibition of DNA synthesis, and DNA strand breaks [2]. Structurally, anthrapyrazoles are similar to mitoxantrone, but their structure has to be modified to reduce the abovementioned side effect. Attempts to reduce the toxicity of anthracyclines have led to the development of various anthrapyrazole derivatives, including teloxantrone (Ci-937, DUP-937, molecule a-60, which is studied in this work), piroxantrone (CI-942, DUP-492, molecule a-58, which is studied in this work), and finally losoxantrone (CI-941, DUP-941), with reduced side effects and increased efficacy in patients with breast cancer. Those three anthrapyrazoles even underwent clinical trials, and in phase II trials, they exhibited significant response rates in women with metastatic breast cancer [3]. Losoxantrone has shown impressive cytotoxic activity on a wide range of tumor cell lines (virtually the same spectrum of antitumor activity as mitoxantrone) with predicted potential to replace anthracyclines through a more favorable therapeutic index [1]. What is more, is that a response rate of 63% in women with metastatic breast cancer was observed in the study conducted by Talbot et al. [5].

The multivariate adaptive regression splines (MARSplines) were presented by Friedman as a method for flexible regression modeling of high dimensional data [6]. This modern machine learning algorithm was successfully applied in a quantitative structure–activity relationship (QSAR), and a quantitative structure–retention relationship (QSRR) modeling approach was applied in studies for drug activity prediction. A MARSplines procedure was used for the development of predictive QSAR models of various compounds with diverse pharmacological activities, such as antitrypanosomal 4-thiazolidinones [7], antispasmodial artemisin compounds [8], pyridine N-oxide derivatives against human severe acute respiratory syndrome [9], or anticancer acridone derivatives [10]. The advantages of the MARS technique were shown, among others, in the case of artemisinin compounds. Namely, it was found that QSAR models determined by the MARS procedure are the most satisfactory predictive models in comparison with some other methods such as multiple linear regression [8]. For abovementioned reasons, the MARSplines algorithm was chosen as a promising tool for a prediction of the antitumor activity of anthrapyrazoles in the present study.

A large set of anthrapyrazole compounds (about 119 derivatives, 73 of which have been studied in the present work) was tested against L1210 murine leukemia in vitro, and P388 leukemia in vivo, by Hollis Showalter et al. [11] In subsequent studies, some of the abovementioned compounds were tested in eight different mouse tumor systems [1]. Moreover, it was found in another study that 12 different anthrapyrazole derivatives inhibited the growth of K562 and K/VP.5 cells [12]. In light of the constant need to develop new anticancer drugs, as well as the high potential of such a large group of anthrapyrazole derivatives studied in the present work, structure–activity studies using modern machine learning algorithms may contribute to achieving better levels of predictivity, thus indicating a potential candidate for further research. The goal of the present work is to create a model predicting the antitumor activity of 73 anthrapyrazole derivatives, as well as to evaluate the usefulness of the MARSplines procedure for QSAR studies.

## 2. Results

More than 2500 molecular descriptors were obtained using Hyperchem and Dragon software, which were used as independent variables to create a model predicting the antitumor activity of 73 anthrapyrazole derivatives ().

### 2.1. Geometry Optimization

**Figure 1:**
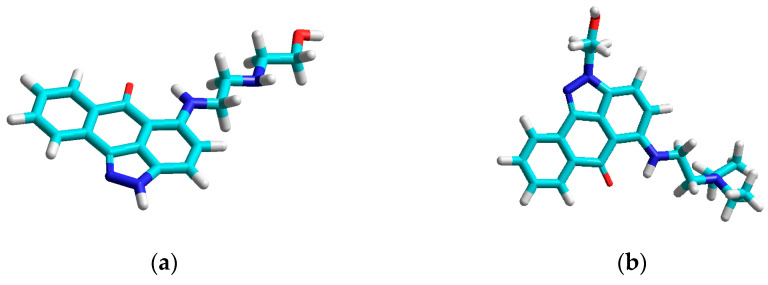

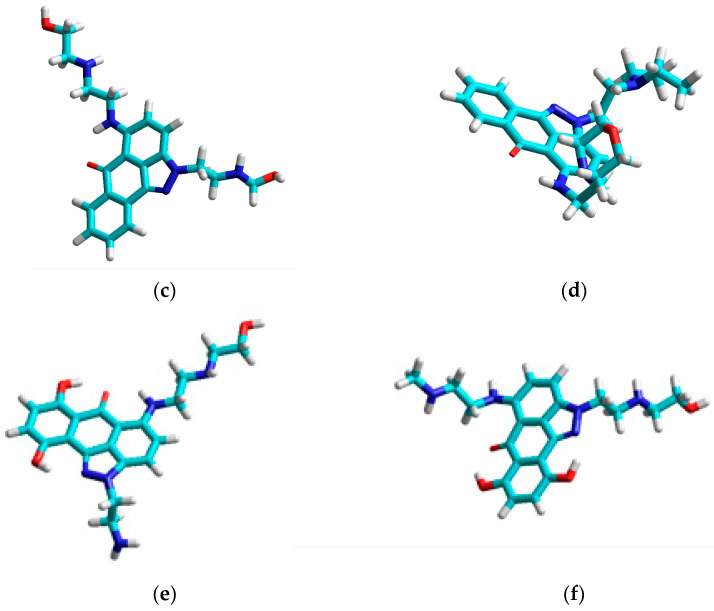
Geometrically optimized structures of selected anthrapyrazole derivatives: (**a**) a-01; (**b**) a-08; (**c**) a-18; (**d**) a-30; (**e**) a-50; (**f**) a-60.

Molecular modeling was performed with 73 derivatives, which were first geometrically optimized. Examples of three-dimensional particle structures with defined geometries are shown in Figure 1.

### 2.2. Statistical Analysis

Completion of an optimal model describing the structure–activity relationship allowed the selection of relevant variables (Mor05s, Mor19m, MATS8e, H1e, ATSC7vk, ATSC1e, SpMax8_Bh(s), Mor21e, Mor13s, R5p, ATSC1s, ATSC8s, RDF135e, and HATS5s) presented in Table 1.

#### 2.2.1. Model Construction and Prediction of pIC_50_ Values

The MARS model, using a considerable set of descriptors as possible predictors, was developed using a training set to describe the antitumor activity denoted as a negative logarithm of the half maximal inhibitory concentration (pIC_50_). The degree of interaction was set at 3, which led to linear, second, and third order splines being incorporated into the model, whereas the maximum number of basis functions was set at 40. Finally, the optimal MARS model was selected on the basis of three validation parameters (R^2^,Q^2^ and MAE). All fourteen descriptors incorporated into the model are characterized in Table 2.

The MARS model is based on several interactions between molecular properties. All of the abovementioned molecular descriptors treated as predictor variables appear in 38 basis functions, which form 23 splines (high-order basis functions) (B_m_). The model starts with the constant function B_1_, and then, in subsequent steps, functions giving the best learning system fit for the current residual are added to the model according to Equation (1):

(1) $${\mathrm{pIC}}_{50}=\overset{22}{\underset{m=1}{\sum }}a_{m}B_{m}$$

The optimal model contains eight single basis functions (B_2_, B_3_, B_8_, B_9_, B_10_, B_21_, B_22_, B_23_), twelve splines that are second-order interactions of two molecular properties (B_4_, B_5_, B_6_, B_7_, B_11_, B_12_, B_13_, B_14_, B_17_, B_18_, B_19_, B_20_), and finally two splines that are third-order interactions of three molecular properties (B_15_, B_16_). All basis functions (B_1_. B_2_…B_23_) and their coefficients a_m_ that comprise the model are shown in Table 3.

As an example of a linear basis function, B_9_ can be considered:

(2) $$(0.42100-\mathrm{Mor}19m{)}_{+}=\{\begin{array}{c} \left(0.42100-\mathrm{Mor}19m\right)\;\mathrm{if}\;\mathrm{Mor}19m<0.42100\;\\ 0\;\mathrm{otherwise} \end{array},$$

What this means, is that the ninth term of Equation (1) is—5.67335 (0.42100 − Mor19m) when Mor19m is lower than 0.42100, and zero when it is smaller than 0.42100. As for an exemplary two-order interaction between molecular properties, B_14_ may be reviewed:

(3) $$\left(\mathrm{MATS}8e-0.07400\right)+\left(\mathrm{RDF}135e-7.04700\right)+\;=\{\begin{array}{c} \left(\mathrm{MATS}8e-0.07400\right)\left(\mathrm{RDF}135e-7.04700\right)\;\mathrm{if}\;\mathrm{MATS}8e>0.07400\;\mathrm{and}\;\mathrm{RDF}135e>7.04700\\ 0\;\mathrm{otherwise} \end{array}$$

What this means is that the fourteenth term of Equation (1) is—8.31766 (MATS8e − 0.07400)(RDF135e − 7.04700) when MATS8e is higher than 0.07400 and RDF135e is higher than 7.04700, but otherwise it is zero.

As mentioned above, 14 of more than 2500 descriptors were incorporated into the MARS model. Their relevance to the MARS model expressed as the number in the basis functions, as well as in their definition, block, and dimensionality which are presented in Table 2. Descriptors describing the molecule’s 3-D geometrical properties (3D-MoRSE descriptors, GETAWAY descriptors, RDF descriptors) emerge in the foreground in the present molecular modeling. The other descriptors are two-dimensional burden eigenvalues and autocorrelations, namely ATS descriptors, which describe how a property is distributed along the topological structure. Out of all the descriptors present in the model, Mor05s and Mor19m descriptors belong to the class of 3D-MoRSE descriptors, and contribute the most to the model, as they appear in the basis functions nine and six times, respectively. The next descriptor is MATS8e, which appears four times in the model, and belongs to the class of 2D autocorrelations, and finally, H1e presents three times as a representative of GETAWAY descriptors. Other descriptors of minor importance for the model (i.e., occurring twice), include ATSC7v, ATSC1e, SpMax8_Bh(s), and R5p. The contributions of ATSC1s, ATSC8s, RDF135e, and HATS5s are much less significant.

#### 2.2.2. Validation of Models and Selection of the Optimal One for Prediction

Using the Multivariate Adaptive Regression Splines nonparametric procedure, 11 QSAR models were created using a different degree of interactions, as well as a different maximum number of basis functions. The coefficients included in the models were determined on the basis of the training group (see Table 1). Following the calculated validation parameters of all models, an optimal model was selected showing the structure–activity relations (degree of interaction 3, number of basis functions 38) with the highest determination coefficient (R^2^) (a perfect correlation was obtained), a cross-validated R^2^ (Q^2^) threshold greater than 0.5 (checking R^2^ for internal validation), and the lowest mean absolute error (MAE). The values of the aforementioned parameters are presented in Table 4.

Moreover, for the optimal MARS model, the extended validation procedure that is typical for QSAR models was applied according to Roy et al. [13] (see Table 5) Considering the above characteristics, the reasonably high predictive power of the established MARS model should be emphasized.

### 2.3. Values of Predicted Data

**Figure 2:**
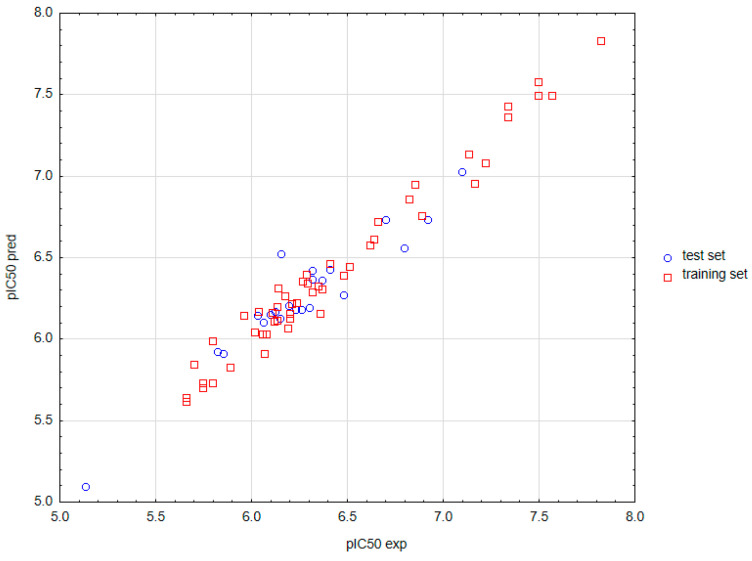
Correlation between the calculated and experimental antitumor data of anthrapyrazoles for the training and test data sets.

**Figure 3:**
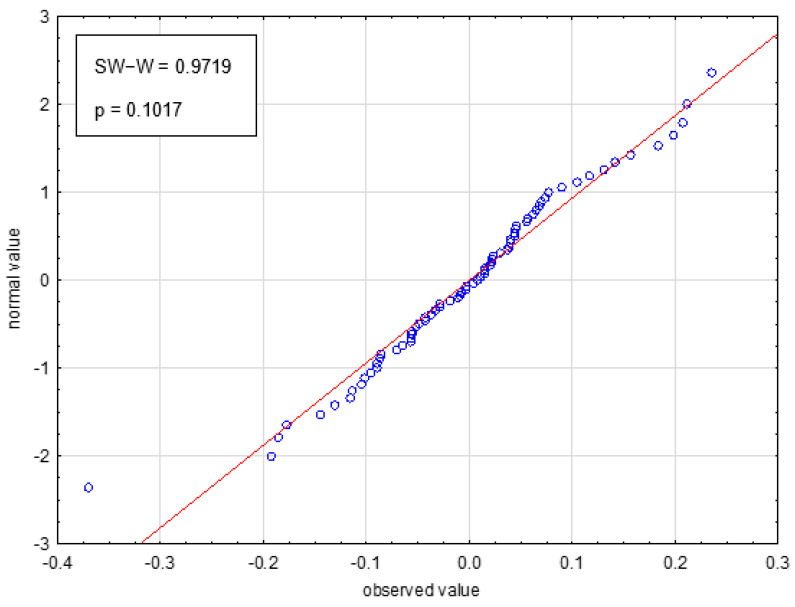
Residual normality plot for the optimal model.

Values of pIC_50_ ($pIC50_{calc}$) obtained on the basis of the constructed model were compared with the experimental data ($pIC50_{exp}$) (see Table S1) and in the scatter plot, where a strong positive relationship is shown (see Figure 2). Moreover, analysis of residuals showed that the residual plot represents a normal distribution (see Figure 3). An elaborated MARS model was also employed for the prediction of antitumor activity against murine leukemia L1210 out of the seven other anthrapyrazole derivatives. This external set was adopted from the literature [1]. It should be noted that the antitumor activity against murine leukemia L1210 has not been reported so far. For more details, see Table S2.

## 3. Discussion

On the basis of the abovementioned validation parameters, namely R^2^, Q^2^, and MAE [13], the optimal predictive and applicative model was selected from the eleven proposed MARS models elaborated in this study, differing in terms of independent variables included, as well as the degree of interactions, and maximum number of basis functions. The interpretation of obtained results begins with a focus on the number and the nature of molecular descriptors present in the model. Fourteen selected descriptors appear in 38 basis functions, which form 23 splines. Predictive descriptors can be divided into the following groups: 3D-MoRSE descriptors, 2D autocorrelations, GETAWAY descriptors, Burden eigenvalues, and RDF descriptors. Descriptors derived from the three-dimensional structure of anthrapyrazole compounds have the highest frequency in repetition (and, in this way, the largest share) in the model (over 68%). This class of geometrical descriptors, which is calculated based on optimized molecular geometry that is obtained by the method of computational chemistry in the current study, comprises 3D-MoRSE descriptors and GETAWAY descriptors. The remaining 32% of the descriptors are calculated from the 2D structure of a molecule (molecular topology).

The 3D-MoRSE Molecular Representation of Structures’ (based on electronic diffraction) descriptors, which have contributed the most to the model percentage-wise (50%), and they comprise the most prominent block of descriptors in the present study. The 3D-MoRSE structure was introduced in 1996 by J.H. Schuur, P. Selzer, and J. Gasteiger in order to encode the 3D structure of a molecule by a fixed number of variables. Each representative of this descriptor block combines the information about the whole molecule structure and its final value, which is derived mostly from short-distance atomic pairs [14]. The 3D-MoRSE descriptors, which are representations of the 3D structure of a molecule, encode features such as molecular weight, van der Waals volume, electronegativities and polarizabilities. In this study, 3D-MoRSE descriptors, weighted by I-state, weighted by mass, and weighted by Sanserson electronegativity, are distinguished. The 3D-MoRSE descriptors cannot describe complex atomic groups or regions with a high or low electron density, or some quantum-chemical properties, but they result in a good model performance when activity variation coincides with variation in interatomic distances due to changes to the bonds’ order and the introduction of new atoms [14].

It was shown that other important factors in predicted antitumor activity (MATS8e, ATSC7v, ATSC1e, ATSC1s, ATSC8s) are 2D autocorrelation descriptors. In general, they explain how the considered property is distributed along the topological structure. An autocorrelation descriptor is a topological descriptor encoding both the molecular structure and physicochemical properties of a molecule [15,16]. The 2D autocorrelations have a share in the optimal model with a percentage of 26.30%.

The next important variables selected belong to GETAWAY (Geometry, Topology, and Atom–Weights Assembly) descriptors (H1e, R5p and HATS5s), which are the block of descriptors that contribute 15.80%. GETAWAY tries to match the 3D molecular geometry provided by the molecular influence matrix and atom relatedness, using topology and chemical information, with the use of various atomic weighting schemes [15].

Another important variable, which is representative of burden eigenvalues with two repetitions in the MARS model, is denoted as SpMax8_Bh(s), which occurs two times in the elaborated MARS model. It belongs to the block of molecular descriptors based on the assumption that the lowest eigenvalues contain contributions from all atoms, and thus, they reflect topology of the molecule [15].

The last parameter, which has been used for modeling and has the smallest frequency, represents RDF (Radial Distribution Function) descriptors, which are based on a radial distribution function. It can be interpreted as the probability distribution of finding an atom in a spherical volume of a radius [15,17].

Efforts to establish mathematical equations for the prediction antitumor activity of anthrapyrazoles also prompt a closer examination and understanding of the mechanism action of these compounds. First of all, anthrapyrazoles with their planar structure can intercalate into DNA. It is well known that compounds that intercalate into DNA stabilize the DNA double helix and increase the temperature at which the DNA is denatured. It is worth noticing that, for a small set of anthrapyrazoles examined in the study, some anthrapyrazole compounds were bound to DNA even more strongly than doxorubicin, the drug hoped, as mentioned before, to be replaced with anthrapyrazoles due to its cardiotoxicity. Anthrapyrazoles not only target DNA, but also interfere with one of the enzymes processing DNA. More specifically, anthrapyrazoles inhibit the decatenation activity of human topoisomerase IIα. This enzyme alters DNA topology by catalyzing the passing of an intact DNA double helix through a transient double-stranded break, which is made in a second helix. Topoisomerase IIα activity is critical for relieving torsional stress that occurs during replication and transcription, and for daughter-strand separation during mitosis. Not only anthrapyrazoles, but also most of the currently used anticancer agents, such as anthracyclines (for instance doxorubicin, mitoxantrone, and etoposide), act as topoisomerase II inhibitors, and their cytotoxicity is a result of the stabilization of a covalent topoisomerase II-DNA intermediate (the cleavable complex). Finally, docking studies on several compounds revealed that the inhibitory activity of anthrapyrazoles is due, in part, to their ability to bind to DNA and structurally similar anthrapyrazoles that can be docked into the doxorubicin-binding pocket on DNA. Moreover, increased binding is associated with increased anthrapyrazole-DNA van der Waals interactions [12].

In the study by Showalter et al. [11] which incorporates the activity data subjected to the current study, the antitumor activity against murine L1210 leukemia in vitro, as well as against P388 leukemia in vivo, was tested over one hundred anthrapyrazole derivatives. Findings of the study indicate that basic side chains at N-2 and C5two to three carbon spaces between proximal and distal nitrogen atoms of the side chain, and A-ring hydroxylations, especially at C-7, contribute to the activity against P388 leukemia growth [11]. Those findings were confirmed by Hartley et al. [18] but the obtained results were not always consistent. On the one hand, the side chains had a greater effect on DNA binding, but on the other hand, the intercalation was affected more by hydroxylation of the A-ring. DNA binding was increased by hydroxylation at C-7 and decreased by hydroxyl groups at any position on the A-ring [18]. Interestingly, in the study by Begleiter et al. [3] anthrapyrazole derivatives showed a broad range of activity for inhibiting topoisomerase II decatenation activity; however, there was no significant correlation with the cytotoxic activity observed. All of the anthrapyrazole analogues examined in this study inhibited the growth of the four cell lines with IC_50_ values that ranged from 0.1 to 45.2 μM, but losoxantrone was the most potent molecule. Structure–activity studies revealed an increase in the cytotoxic activity with the presence of a tertiary amine in the basic side chain at N-2, in comparison with a secondary amine in the same position for the majority of examined derivatives, but only in the case of the absence of a basic side chain at the C-5 position. Other structural alternations, such as a chlorine substituent on the basic side chain at N-2, moving the position of a chlorine substituent from C-5 to C-7, or introducing a basic side chain at C-5, did not have a consistent effect on cytotoxic activity. The authors of this study suggested that the ability of the analogues to bind to DNA by alkylation does not contribute significantly to the antitumor activity of the anthrapyrazoles [3]. A study by Liang et al. [12] confirmed the abovementioned results. Namely, cell growth inhibition by anthrapyrazoles was not well-correlated with the inhibition of topoisomerase IIα catalytic activity, which suggests that the anthrapyrazole derivatives examined in this study did not act solely by inhibiting the catalytic activity of topoisomerase II. Moreover, the authors showed that hydrogen-bond donor interactions and electrostatic interactions with the protonated amino side chains of the anthrapyrazoles led to high cell growth inhibitory activity [12].

The abovementioned studies demonstrated that structural changes on the basic side chain at N-2, and at C-5, C-7, can have a considerable impact on the cytotoxic activity of anthrapyrazoles as well as on topoisomerase II inhibition. Those results that are still inconsistent, may even, to small extent, help to understand the role of descriptors incorporated into the optimal MARS model. In the present study, descriptors derived from the three-dimensional structure of anthrapyrazole compounds comprise the largest share in the model, alongside the most prominent 3D-MoRSE descriptors, with values that are very sensitive to any conformational change in the molecule, and GETAWAY descriptors encoding information about the influence that each atom has in determining the whole shape of the molecule. In this light, the antitumor activity difference of the anthrapyrazoles studied, is presumably a result of the interatomic distances’ changes, or the introduction of new atoms at N-2 and C-5, C-7. The obtained data indicate that parameters based on the molecular geometry and physicochemical properties, the reflection of molecular topology, and finally, the distance distribution of the compounds, are of the greatest importance for the antitumor activity of the anthrapyrazole derivatives.

It should be emphasized that the MARS model has been expanded upon in the present study, in order to predict the antitumor activity of 73 anthrapyrazole compounds, so that it is able to describe more than 96% of the variance in the experimental activity. Good predictive properties of the model were confirmed by an extensive validation procedure, which is characteristic of QSAR models. Several validation parameters were calculated. Among others, cross-validated R^2^ was checked for internal validation, mean absolute error was calculated, and predictability, as well as precision and accuracy, were also assessed. It should be noted that all tested parameters met the acceptance criteria [13] listed in Table 5. Moreover, the MARS model that was created may be successfully employed for the prediction of the antitumor activity of anthrapyrazole compounds. Its applicative value was confirmed by an external set of seven molecules with the predicted pIC_50_ listed in Table S2.

Searching the literature, it is still easier to come across QSAR analysis based on multiple linear regression than multivariate adaptive regression splines. Nevertheless, MARSplines procedure is one of the modern machine learning algorithms with numerous advantages that are emphasized in this work. Its usefulness was confirmed for QSAR studies for predicting the antimalarial activity of dihydroartemisinin derivatives by Nguyen-Cong et al. [8], antitumor activity of acridone derivatives by Koba and Bączek [10], or anti-HIV activities of thiazolylthiourea derivatives by Alamdari et al. [19]. The present study strongly supports the idea of promoting the MARSplines technique in QSAR analysis; however, it should be considered that, given the multitude of possible datasets and descriptors available, various options for MARSplines analysis, as well as other modern machine learning algorithms with their numerous advantages, in a particular case or other regression procedure, may show a better performance. This trend is visible in the study by Kryshchyshyn et al. [7] where four machine learning algorithms, namely, Random forest regression, Stochastic gradient boosting, Multivariate adaptive regression splines, and Gaussian processes regression, were studied to reach better levels of predictivity. Finally, in the case of predicting the antitrypanosomal activity of 4-thiazolidinones, a model developed only with the Random forest and Gaussian processes regression algorithms had good predictive ability. In light of this, there is no universal regression method, but different studies prove that modern machine algorithms are worth exploring.

In sum, the obtained model can be successfully used for in silico studies in order to find new compounds with promising antitumor activity. On the one hand, it can be assumed that the presented approach has some limitations as a restriction to the chemical domain of the training set, especially A-ring hydroxylation at C-7,10, and a necessity to follow whole procedure of geometry optimization and descriptor calculation; however, on the other hand, it should be taken into consideration that there are a multitude of combinations of different possible substituents at N-2 and C-5 in the anthrapyrazole ring (some of them were tested so far and some of them were considered promising). What is more, is that the semi-empirical method AM1 for geometry optimization and the overall process of descriptor generation are fast, which speaks for a routine application of a presented MARSplines approach for QSAR studies. For the abovementioned reasons, the expanded MARSplines procedure may become a part of the process of drug design, largely as it may be useful in the selection of the new anticancer compounds of anthrapyrazoles for the synthesis and in vitro testing on various cancer cell lines.

## 4. Materials and Methods

### 4.1. Anthrapyrazole Derivatives

The conducted analyses were founded on 73 compounds of anthrapyrazole derivatives (Anthra [1,9-cd]pirazol-6(2H)-on), differing in both chemical structure and antitumor activity, as shown in Table 6. The data concerning the antitumor activity of anthrapyrazoles against the L1210 murine leukemia cell line, tested in vivo, and expressed as IC_50_, were obtained from the literature [11].

### 4.2. Geometry Optimization and Structural Descriptors

The initial optimization of the geometric structures of the analyzed particles, with the use of specialized HyperChem Release 8.0 (Hypercube Inc., Gainesville, FL, USA) software, was performed using the built-in Molecular Mechanic Force Field (MM+) procedure, taking into account the adequacy of the principles of quantum mechanics. In the next step, the proper optimization was achieved using the Semi-Empirical Molecular Method AM1, with the utilization of the Polak–Ribiere algorithm. The gradient norm limit applied for the calculations was 0.01 kcal (Å⋅mol)^−1^, and the maximum possible number of cycles was set to 32,000. Finally, HyperChem, as well as Dragon 7 (Talete, Milano, Italy) software, were used to obtain molecular descriptors for all studied structures, using previously optimized molecules. In total, 2554 descriptors were calculated, mainly by using Dragon software. In the next stage of the study, the obtained descriptors were subjected to MARSplines analysis. Descriptors calculated by Dragon include 29 logical molecular descriptor blocks: constitutional indices, ring descriptors, topological indices, walk and path counts, connectivity indices, information indices, 2D matrix-based descriptors, 2D autocorrelations, burden eigenvalues, P_VSA-like descriptors, ETA indices, edge adjacency indices, geometrical descriptors, 3D matrix-based descriptors, 3D autocorrelations, RDF descriptors, 3D-MoRSE descriptors, WHIM descriptors, GETAWAY descriptors, randic molecular profiles, functional group counts, atom-centered fragments, atom-type E-state indices, CATS 2D, 2D atom pairs, 3D atom pairs, charge descriptors, molecular properties, and drug-like indices [20].

### 4.3. Statistical Analysis

Statistical analysis was carried out using Statistica 13.3 software (StatSoft, Cracow, Poland), introducing the data obtained in the previously performed molecular modeling. The analysis used the following variables: descriptors describing molecular properties of a particle, and the values of the negative decimal logarithm of the IC_50_ describing the biological activity against the L1210 murine leukemia cell line tested in vitro, obtained from the literature data. The whole group of compounds was divided into a training and test set on the basis of random sample selection in STATISTICA 13.3 Data Miner (StatSoft, Cracow, Poland). Raw data, consisting of 2554 descriptors (independent variables) and negative decimal logarithm values of the IC_50_ (pIC_50_, dependent variable), were subjected to a process of standardization and pre-selection. The selection consisted of removing the variables that did not show variability. The analyses were performed at the 5% significance level (α = 0.05). The multivariate adaptive regression splines procedure was used to build eleven different models. Pearson’s correlation coefficient was used in the analysis of the correlation of variables. J. Guilford’s classification was used for the interpretation of the results. The analysis of three validation parameters, providing minimal but sufficient information about model performance (R^2^, Q^2^, MAE) [13,21], and is explained in Section 4.4, allowed for the selection of an optimal theoretical model aimed at predicting the pIC_50_ value for each of the considered derivatives.

### 4.4. MARSplines Analysis

Multivariate Adaptive Regression Splines (MARSplines), performed with the use of Statistica 13.3, is an adaptive procedure for regression. The specification of MARSplines analysis is shown in Table 7. It is very useful, especially to solve high-dimensional problems, such as a large number of inputs. Moreover, it is used to solve both regression and classification problems, and does not require assumptions about the functional relationship between independent (input) and dependent (output) data. This relationship is modeled with the use of base functions and a set of coefficients generated solely on the basis of data [6,22].

The basis functions in MARS model are single truncated spline functions, or an interaction of a few spline functions, and they consist of a left-sided and right-sided segment (reflected pair) (Equation (4)). The subscript “+” means the positive part, thus:

(4) $${\left(x-t\right)}_{+}=\{\begin{array}{c} x-t,\;\mathrm{if}\;x>t,\\ 0,\;\mathrm{otherwise}, \end{array}\;{\left(t-x\right)}_{+}=\{\begin{array}{c} t-x,\;\mathrm{if}\;x<t\\ 0,\;\mathrm{otherwise}. \end{array}$$

Each function is piecewise linear, with a knot at value t (the so-called linear spline). Those reflected pairs are formed for each input X_j_, with knots at each observed value x_ij_ of that input. That is why the collection of basis functions is as follows:

(5) $$C={\{{\left(X_{j}-t\right)}_{+},{\left(t-X_{j}\right)}_{+}}_{\begin{array}{c} t\in \;\{x_{1j},x_{2j},\ldots ,x_{\mathrm{Nj}}\}\\ j=1,2\ldots ,p \end{array}}$$

If all of the input values are distinct, there are 2Np basis functions altogether. Although each basis function depends only on a single X_j_, it is still considered as a function over the entire input space IR^p^.

The model–building approach is similar to a forward stepwise linear regression, but instead of using the original inputs, functions from set C and their products are used, so the model is as follows:

(6) $$f\left(X\right)=\beta_{0}+\overset{M}{\underset{m-1}{\sum }}\beta_{m}h_{m}\left(X\right)$$

where each h_m_(X) is a function in C, or a product of two or more such functions. During each iteration, the best reflected pair is chosen and all possible predictors, as well as corresponding knot locations, are evaluated. As a result of each iteration, the so-called interactions may be introduced if this improves the model. The building process stops when a user-defined maximum number of basis functions is reached; however, it should be emphasized that, during model building, a global model usually overfits the training data. That is why, in the next step, a pruning procedure based on generalized cross-validation (GCV) is applied, which leads to exclusion functions that receive the lowest contribution from the model. The GCV parameter, comprising a penalty for the model complexity, is an adjusted residual sum of squares used to prevent the occurrence of an excessive number of spline functions in the final model.

### 4.5. Model Validation

Elaborated models underwent a process of validation in the terms of the determination coefficient, cross validated determination coefficient, and mean absolute error, in order to select the optimal MARS model suitable for the prediction of the antitumor activity of the anthrapyrazoles studied [13].

(7) $$R^{2}=1-\frac{\sum^{\;}{\left(Y_{\mathrm{obs}}-Y_{\mathrm{cal}}\right)}^{2}}{\sum^{\;}{\left(Y_{\mathrm{obs}}-{\;\bar{Y}}_{\mathrm{training}}\right)}^{2}}$$

The determination coefficient R^2^ (Equation (7)) measures the variation of observed data with the predicted data. A perfect correlation is observed when the R^2^ reaches the maximum possible value (i.e., 1. Y_obs_ denotes the observed response values for the training set, and Y_calc_ denotes the calculated response values for the training set of compounds. Y_training_ is the mean observed response of the training set compounds [13]).

(8) $$Q^{2}\left({\mathrm{orQ}}_{\mathrm{LOO}}^{2}\right)=1-\frac{\sum^{\;}{\left(Y_{\mathrm{obs}\left(\mathrm{training}\right)}-Y_{\mathrm{pred}\left(\mathrm{training}\right)}\right)}^{2}}{\sum^{\;}{\left(Y_{\mathrm{obs}\left(\mathrm{training}\right)}-{\;\bar{Y}}_{\left(\mathrm{training}\right)}\right)}^{2}}$$

Cross-validated R^2^ (Q^2^), presented in Equation (8), is checked for internal validation. Y_obs_(training) is the observed response, and Y_pred_ (training) is the predicted response of the training set molecules based on the leave-one-out (LOO) technique. The generally accepted threshold value of Q^2^ is 0.5 [13].

(9) $$\mathrm{MAE}=\frac{\sum^{\;}\left|Y_{\mathrm{obs}}-Y_{\mathrm{pred}}\right|}{n}$$

The mean absolute error (MAE) (Equation (9)) is also recognized as the average absolute error (AAE). Generally, it is regarded as a superior index of errors in the context of predictive modeling studies. Due to the involvement of the squared term of the prediction errors in the expression of RMSE, the variance of errors may be influenced by a set of data. That is because squaring the higher prediction error values have more weight than the lower errors in the formalism of the root mean square error (RMSE), whereas MAE provides an equal weight to all errors; thus, MAE is considered to be a simpler and more straightforward determinant of prediction errors [13].

For the optimal MARS model following validation, the parameters as follows: R^2^, Q^2^, Q_F1_^2^, Q_F2_^2^, Q_F3_^2^, CCC, ∆r_m_^2^, $\bar{r_{m}^{2}}$, PRES, SDEP, and MAE, were calculated according to Roy et al. [13].

## 5. Conclusions

A quantitative structure–activity relationship study was applied to a large set of anthrapyrazole compounds presenting antitumor activity against murine leukemia L1210. The approach of MARSplines was employed for prediction purposes, and was able to describe more than 96% of the variance in the experimental activity. This study has shown that fourteen parameters appearing in the statistically significant and extensively validated MARS model (i.e., descriptors belonging to 3D-MoRSE, 2D autocorrelations, GETAWAY, burden eigenvalues and RDF descriptors) significantly affect the antitumor activity of anthrapyrazole compounds. Moreover, this study confirmed the benefit of using the modern machine learning algorithm, namely, the MARSplines procedure, because the elaborated flexible model was also used in the prediction of antitumor activity against murine leukemia L1210 using an external set of seven anthrapyrazole compounds. Finally, in light of the potential laying in such a large set of anthrapyrazole compounds, which still may be tested on various cell lines, and the high predictive power of the MARS model, the MARSplines procedure may be useful in the selection of the anticancer compounds of anthrapyrazoles for future clinical studies.

## Figures and Tables

**Table 1:** Values of significant molecular descriptors for the tested anthrapyrazole derivatives.

Compound	Set	Descriptors													
		Mor05s	Mor19m	MATS8e	H1e	ATSC7v	ATSC1e	SpMax8_Bh(s)	Mor21e	Mor13s	R5p	ATSC1s	ATSC8s	RDF135e	HATS5s
a-01	training	−23.248	0.14	0.071	1.868	8.747	0.089	3.701	−1.038	−3.106	0.355	10.915	24.843	3.716	0.885
a-02	test	−24.151	0.35	0.086	2.449	10.376	0.063	3.729	−1.668	−2.011	0.431	7.562	17.708	2.312	0.888
a-03	test	−25.044	0.312	0.088	1.887	10.229	0.072	3.805	−1.273	−3.232	0.352	9.636	26.817	4.833	0.696
a-04	training	−26.013	0.527	0.093	2.415	11.909	0.062	3.826	−1.909	−1.983	0.42	7.486	19.947	2.33	0.731
a-07	training	−28.355	0.374	0.136	2.266	12.334	0.108	3.868	−1.422	−3.935	0.34	14.861	70.152	8.044	0.59
a-08	training	−29.427	0.556	0.173	2.494	14.042	0.067	3.892	−1.992	−2.491	0.393	9.462	63.182	6.066	0.623
a-14	test	−27.108	0.38	0.081	2.352	13.021	0.09	3.868	−1.49	−4.166	0.356	12.928	53.706	7.109	0.56
a-15	training	−24.585	0.405	0.087	2.499	10.664	0.072	3.731	−0.942	−2.711	0.462	9.69	34.523	0	1.068
a-16	test	−27.47	0.41	0.104	2.485	12.554	0.108	3.93	−1.01	−5.516	0.43	14.861	50.769	2.714	0.979
a-17	training	−26.332	0.399	0.045	2.651	13.246	0.09	3.91	−0.998	−4.039	0.463	12.928	48.529	2.581	0.748
a-18	training	−27.16	0.354	0.051	2.486	14.206	0.117	4.11	−1.333	−4.656	0.372	15.025	49.723	9.628	0.857
a-19	training	−27.182	0.593	0.084	2.453	15.132	0.071	3.913	−1.856	−4.49	0.397	10.351	43.961	5.776	0.78
a-20	training	−30.264	0.711	0.074	2.685	16.896	0.084	3.943	−2.188	−2.689	0.513	10.256	41.257	8.767	0.675
a-21	training	−30.336	0.887	−0.015	2.454	18.916	0.093	3.892	−2.927	−1.602	0.439	9.089	37.67	14.127	0.535
a-23	training	−28.063	0.473	0.052	2.395	15.141	0.079	3.892	−1.916	−3.363	0.473	9.555	42.176	3.359	0.664
a-24	training	−28.566	0.547	0.075	2.397	16.123	0.079	3.892	−2.428	−2.117	0.456	8.749	39.386	6.171	0.6
a-25	training	−28.039	0.772	0.062	2.431	17.093	0.084	3.892	−1.927	−3.76	0.447	10.256	40.357	5.936	0.665
a-26	test	−28.873	0.779	0.086	2.44	18.887	0.093	3.892	−2.847	−1.14	0.526	9.581	35.763	10.198	0.721
a-27	training	−32.526	0.886	0.054	2.46	20.027	0.099	3.892	−3.152	−2.312	0.496	10.073	41.721	15.563	0.608
a-28	training	−31.513	0.772	−0.034	2.59	21.69	0.105	3.892	−3.454	−0.694	0.575	10.551	43.578	19.845	0.613
a-29	training	−35.189	1.108	0.022	2.504	25.218	0.122	3.941	−4.494	0.073	0.54	11.901	44.571	23.851	0.586
a-30	test	−29.895	0.984	0.074	2.711	18.211	0.069	3.892	−2.825	−3.798	0.571	7.221	34.729	4.623	0.685
a-31	test	−32.648	1.182	0.088	2.692	18.629	0.09	3.892	−2.855	−1.78	0.566	8.368	34.604	4.618	0.639
a-32	training	−37.679	0.603	0.07	2.84	23.509	0.063	4.302	−3.52	−2.193	0.555	10.118	61.697	2.066	0.602
a-33	training	−31.756	0.295	−0.15	1.854	7.964	0.15	3.907	−0.585	−6.758	0.325	19.422	68.778	0	0.778
a-34	training	−32.952	0.272	−0.084	1.962	9.944	0.173	4.222	−0.896	−7.17	0.355	21.396	65.841	4.312	0.899
a-35	training	−34.132	0.496	−0.082	2.486	11.745	0.09	3.942	−1.561	−5.943	0.429	12.554	50.107	5.362	0.949
a-36	training	−36.292	0.493	−0.047	2.613	14.261	0.089	4.279	−1.313	−5.809	0.423	13.378	57.153	3.503	0.845
a-38	training	−35.884	0.327	0.03	2.49	13.45	0.186	4.35	−1.215	−7.443	0.375	21.7	96.608	7.612	0.876
a-40	training	−35.247	0.336	−0.005	2.396	10.119	0.194	4.342	−0.67	−7.823	0.364	27.496	130.412	0	0.885
a-41	training	−36.566	0.293	0.019	2.379	12.094	0.218	4.352	−1.021	−7.791	0.354	29.098	127.386	7.555	0.807
a-42	training	−36.299	0.373	0.052	2.445	12.008	0.152	4.281	−1.172	−7.005	0.324	19.476	115.944	1.162	0.692
a-43	training	−37.466	0.485	0.052	2.58	13.9	0.128	4.279	−1.574	−6.836	0.419	18.037	111.512	4.311	0.833
a-44	training	−38.067	0.711	0.036	2.823	13.123	0.178	4.346	−1.597	−7.37	0.535	20.604	115.411	0	1.067
a-46	training	−40.983	0.422	0.034	2.466	16.838	0.191	4.376	−1.732	−7.572	0.369	26.156	131.348	11.288	0.83
a-47	test	−34.614	0.389	−0.101	2.466	10.872	0.166	4.339	−0.836	−7.493	0.38	24.127	106.027	0	0.873
a-48	training	−34.595	0.45	−0.09	2.484	12.098	0.155	4.343	−1.003	−7.772	0.37	23.038	115.094	0	0.866
a-49	test	−34.259	0.372	−0.064	2.438	11.805	0.147	4.281	−1.068	−7.085	0.368	18.738	96.646	0.712	0.82
a-50	training	−37.076	0.338	−0.056	2.383	12.827	0.189	4.351	−0.811	−7.866	0.355	25.724	102.909	8.225	0.782
a-51	test	−38.93	0.529	−0.085	2.593	14.213	0.177	4.35	−0.971	−7.42	0.451	24.541	110.401	2.928	1.43
a-52	test	−39.941	0.441	−0.064	2.404	15.776	0.126	4.331	−1.487	−6.097	0.372	16.891	97.497	10.951	0.628
a-53	test	−36.787	0.433	−0.133	2.481	14.405	0.177	4.354	−1.006	−8.223	0.363	24.541	84.423	7.287	0.858
a-54	test	−39.871	0.503	−0.151	2.419	17.328	0.116	4.336	−1.735	−6.213	0.358	16.214	74.598	13.241	0.756
a-55	training	−36.81	0.361	−0.043	2.5	14.185	0.169	4.308	−1.119	−7.462	0.376	20.409	83.802	6.619	0.863
a-56	training	−34.217	0.218	−0.08	2.735	13.043	0.189	4.354	−0.752	−9.059	0.476	25.724	93.711	2.594	0.994
a-57	training	−36.624	0.562	−0.074	2.586	14.298	0.177	4.356	−0.99	−10.128	0.414	24.541	102.629	3.705	1.362
a-60	training	−33.756	0.421	−0.054	2.572	14.011	0.169	4.302	−0.952	−8.742	0.416	20.409	84.468	4.29	1.368
a-62	training	−37.503	0.552	−0.048	2.772	15.019	0.212	4.359	−1.275	−8.56	0.43	27.21	90.663	3.006	0.789
a-63	training	−41.791	0.386	−0.072	2.813	16.385	0.199	4.358	−1.074	−9.141	0.5	25.97	97.975	2.933	0.898
a-64	test	−41.408	0.429	−0.024	2.67	17.542	0.234	4.447	−1.311	−9.127	0.436	32.045	92.108	8.337	1.114
a-65	training	−42.394	0.647	−0.069	2.737	18.936	0.22	4.445	−1.996	−8.813	0.484	30.676	102.443	9.966	0.994
a-66	training	−35.171	0.471	−0.031	2.571	14.998	0.149	4.297	−1.291	−8.145	0.405	18.91	79.856	7.264	1.18
a-67	training	−37.34	0.443	−0.033	2.746	16.838	0.126	4.296	−2.132	−8.091	0.507	17.382	73.971	5.859	0.842
a-68	training	−37.247	0.848	−0.033	2.849	16.092	0.172	4.356	−1.65	−8.684	0.512	19.233	77.96	2.225	0.842
a-69	training	−35.64	0.589	−0.089	2.618	15.742	0.14	4.267	−1.564	−7.446	0.426	17.569	78.636	7.216	0.946
a-70	test	−36.907	0.519	−0.048	2.682	15.457	0.186	4.358	−1.247	−8.227	0.395	24.211	85.133	10.285	0.744
a-71	training	−39.599	0.429	−0.055	2.558	17.935	0.146	4.348	−1.844	−6.618	0.381	18.312	84.952	13.548	0.808
a-73	training	−39.446	0.471	−0.062	2.605	18.474	0.129	4.297	−1.592	−7.497	0.415	17.763	82.352	4.101	0.769
a-74	training	−38.284	0.554	−0.131	2.505	15.558	0.165	4.353	−1.035	−8.24	0.401	23.364	100.733	11.198	0.861
a-76	training	−38.074	0.387	−0.038	2.597	16.406	0.191	4.357	−1.39	−6.331	0.411	25.148	83.897	8.133	0.984
a-77	training	−35.23	0.447	−0.064	2.558	13.577	0.128	4.285	−0.952	−6.533	0.464	17.289	79.755	0	0.918
a-78	training	−36.941	0.529	−0.06	2.475	14.84	0.117	4.289	−1.303	−7.397	0.416	16.659	88.64	2.311	1.377
a-79	test	−32.38	0.413	−0.033	2.727	15.563	0.149	4.303	−1.113	−7.931	0.463	18.91	77.174	7.047	0.88
a-80	test	−35.172	0.604	−0.18	2.487	13.832	0.117	4.312	−1.267	−7.558	0.463	16.659	74.551	2.789	1.292
a-81	test	−38.719	0.681	−0.169	2.511	15.108	0.108	4.315	−1.611	−7.898	0.415	15.991	82.931	4.808	1.146
a-82	test	−35.243	0.672	−0.127	2.766	15.826	0.137	4.323	−1.786	−8.211	0.488	18.164	72.004	0.053	0.897
a-83	training	−36.32	0.495	−0.062	2.546	15.014	0.108	4.286	−1.608	−8.139	0.532	15.974	73.76	0.419	0.896
a-84	test	−36.382	0.473	−0.039	2.465	16.024	0.089	4.26	−1.908	−6.312	0.465	12.755	67.073	5.692	0.813
a-86	training	−38.16	0.486	−0.034	2.499	17.02	0.126	4.301	−1.772	−7.501	0.447	17.382	71.601	6.725	0.797
a-87	test	−38.523	0.577	−0.021	2.535	18.877	0.089	4.257	−2.65	−5.673	0.481	13.34	61.78	18.608	0.73
a-88	test	−39.825	0.429	−0.048	2.644	17.927	0.146	4.353	−2.036	−7.398	0.406	18.312	82.663	18.698	0.771
a-90	training	−39.406	0.784	0.02	2.675	18.546	0.166	4.352	−1.824	−6.002	0.486	24.308	118.33	17.894	0.78
a-91	training	−40.792	1.003	0.048	2.645	20.3	0.095	4.302	−2.484	−5.435	0.529	17.575	106.766	18.072	0.75

**Table 2:** Selected descriptors and the number of times they appeared in the basis functions of the MARS model.

Symbol	Definition	Block	Dimensionality	Number in the Basis Function
Mor05s	signal 05/weighted by I-state	3D-MoRSE descriptors	3D	9
Mor19m	signal 19/weighted by mass	3D-MoRSE descriptors	3D	6
MATS8e	Moran autocorrelation of lag 8 weighted by Sanderson electronegativity	2D autocorrelations	2D	4
H1e	H autocorrelation of lag 1/weighted by Sanderson electronegativity	GETAWAY descriptors	3D	3
ATSC7v	Centred Broto–Moreau autocorrelation of lag 7 weighted by van der Waals volume	2D autocorrelations	2D	2
ATSC1e	Centred Broto–Moreau autocorrelation of lag 1 weighted by Sanderson electronegativity	2D autocorrelations	2D	2
SpMax8_Bh(s)	largest eigenvalue n. 8 of Burden matrix weighted by I-state	Burden eigenvalues	2D	2
Mor21e	signal 21/weighted by Sanderson electronegativity	3D-MoRSE descriptors	3D	2
Mor13s	signal 13/weighted by I-state	3D-MoRSE descriptors	3D	2
R5p	R autocorrelation of lag 5/weighted by polarizability	GETAWAY descriptors	3D	2
ATSC1s	Centred Broto–Moreau autocorrelation of lag 1 weighted by I-state	2D autocorrelations	2D	1
ATSC8s	Centred Broto–Moreau autocorrelation of lag 8 weighted by I-state	2D autocorrelations	2D	1
RDF135e	Radial Distribution Function—135/weighted by Sanderson electronegativity	RDF descriptors	3D	1
HATS5s	leverage-weighted autocorrelation of lag 5/weighted by I-state	GETAWAY descriptors	3D	1

**Table 3:** The functions of the basis splines.

B_m_	Definition	a_m_
B_1_	1	7.00228
B_2_	(Mor05s + 28.56600)_+_	−0.41345
B_3_	(−28.56600 − Mor05s)_+_	−0.10460
B_4_	(ATSC7v − 12.33400) _+_ (Mor05s + 28.56600)_+_	0.29808
B_5_	(12.33400 − ATSC7v) _+_ (Mor05s + 28.56600)_+_	0.11583
B_6_	(−28.56600 − Mor05s) _+_ (R5p − 0.37500)_+_	0.98096
B_7_	(−28.56600 − Mor05s) _+_(0.37500 − R5p) _+_	3.57380
B_8_	(Mor19m − 0.42100) _+_	−1.63111
B_9_	(0.42100 − Mor19m)_+_	−5.67335
B_10_	(MATS8e − 0.07400)_+_	−14.65355
B_11_	(15.99100 − ATSC1s)_+_ (0.07400 − MATS8e)_+_	−6.03111
B_12_	(70.15200 − ATSC8s)_+_ (0.07400 − MATS8e)_+_	0.92668
B_13_	(−28.56600 − Mor05s)_+_ (H1e − 2.54600)_+_	−0.53694
B_14_	(MATS8e − 0.07400)_+_ (RDF135e − 7.04700)_+_	−8.31766
B_15_	(SpMax8_Bh(s) − 4.32300)_+_ (−28.56600 − Mor05s)_+_ (0; 2.54600 − H1e)_+_	−16.59500
B_16_	(4.32300 − SpMax8_Bh(s))_+_ (−28.56600 − Mor05s)_+_ (2.54600 − H1e)_+_	−0.64411
B_17_	(Mor19m − 0.42100)_+_ (Mor21e + 1.26700)_+_	−19.90208
B_18_	(Mor19m − 0.42100)_+_ (−1.26700 − Mor21e)_+_	−0.88179
B_19_	(Mor19m − 0.42100)_+_ (Mor13s + 6.31200)_+_	0.33453
B_20_	(Mor19m − 0.42100)_+_ (−6.31200 − Mor13s)_+_	0.65372
B_21_	(0.85700 − HATS5s)_+_	1.71725
B_22_	(ATSC1e − 0.11600)_+_	6.68741
B_23_	(0.11600 − ATSC1e)_+_	6.15634

**Table 4:** Values of validation parameters of models obtained with the MARSplines procedure (the optimal model marked in yellow).

Degree of Interaction	Number of Basis Functions	R^2^	Q^2^	MAE
1	6	0.5291	−0.1525	0.2622
	16	0.8288	0.5787	0.1709
	21	0.9277	0.8706	0.1133
	21	0.9185	0.8807	0.1230
2	6	0.4691	0.1343	0.2819
	16	0.8649	0.7480	0.1616
	33	0.9328	0.9311	0.1096
3	6	0.4691	0.1343	0.2819
	26	0.8649	0.7480	0.1616
	38	0.9617	0.9016	0.0772
	40	0.9532	0.9033	0.0897

**Table 5:** Values of validation parameters of the optimal MARS model.

Parameter [13]	Value	Threshold [13]	Meaning [13]
$R^{2}=1-\frac{\sum^{\;}{\left(Y_{\mathrm{obs}}-Y_{\mathrm{cal}}\right)}^{2}}{\sum^{\;}{\left(Y_{\mathrm{obs}}-{\bar{Y}}_{\mathrm{training}}\right)}^{2}}$	0.9617	~1 (1 means perfect correlation)	It measures the variation of observed data with the predicted ones.
$Q^{2}\left({\mathrm{orQ}}_{\mathrm{LOO}}^{2}\right)=1-\frac{\sum^{\;}{\left(Y_{\mathrm{obs}\left(\mathrm{training}\right)}-Y_{\mathrm{pred}\left(\mathrm{training}\right)}\right)}^{2}}{\sum^{\;}{\left(Y_{\mathrm{obs}\left(\mathrm{training}\right)}-{\bar{Y}}_{\left(\mathrm{training}\right)}\right)}^{2}}$	0.9016	≥0.5	Cross-validated R^2^ (Q^2^) checked for internal validation.
$Q_{F1}^{2}=1-\frac{\sum^{\;}{\left(Y_{\mathrm{obs}\left(\mathrm{test}\right)}-Y_{\mathrm{pred}\left(\mathrm{test}\right)}\right)}^{2}}{\sum^{\;}{\left(Y_{\mathrm{obs}\left(\mathrm{test}\right)}-{\bar{Y}}_{\left(\mathrm{training}\right)}\right)}^{2}}$	0.9119	≥0.5	A measure of correlation between the observed and predicted data of the test set.
$Q_{F2}^{2}=1-\frac{\sum^{\;}{\left(Y_{\mathrm{obs}\left(\mathrm{test}\right)}-Y_{\mathrm{pred}\left(\mathrm{test}\right)}\right)}^{2}}{\sum^{\;}{\left(Y_{\mathrm{obs}\left(\mathrm{test}\right)}-{\bar{Y}}_{\left(\mathrm{test}\right)}\right)}^{2}}$	0.90163	≥0.5	Almost equal or closer values of Q^2^_(F2)_ and Q^2^_(F1)_ infer that the training set mean lies in the close propinquity to that of the test set.
$Q_{F3}^{2}=1-\frac{[\sum^{\;}{\left(Y_{\mathrm{obs}\left(\mathrm{test}\right)}-Y_{\mathrm{pred}\left(\mathrm{test}\right)}\right)}^{2}]/n_{\mathrm{test}}}{\left[\sum^{\;}{\left(Y_{\mathrm{obs}\left(\mathrm{train}\right)}-{\bar{Y}}_{\left(\mathrm{train}\right)}\right)}^{2}\right]/n_{\mathrm{train}}}$	0.7959	≥0.5	It measures the model predictability.
$\mathrm{CCC}=\frac{2\sum_{i=1}^{n}\left(x_{i}-\bar{x}\right)\left(y_{i}-\bar{y}\right)}{\sum_{i=1}^{n}{\left(x_{i}-\bar{x}\right)}^{2}+\sum_{i=1}^{n}\left(y_{i}-\bar{y}\right)+n\left(\bar{x}-\bar{y}\right)}$	0.9496	~1	Concordance correlation coefficient (CCC) measures both precision and accuracy, detecting the distance of the observations from the fitting line and the degree of deviation of the regression line from that passing through the origin, respectively.
$\bar{r_{m}^{2}}=\frac{\left(r_{m}^{2}+{r^{\prime }}_{m}^{2}\right)}{2}$$\mathrm{and}\;\Delta r_{m}^{2}=\left|r_{m}^{2}-{r^{\prime }}_{m}^{2}\right|$, $\mathrm{where}\;r_{m}^{2}=r_{}^{2}\times \left(1-\sqrt{r_{}^{2}-}r_{0}^{2}\right)$ ${r^{\prime }}_{m}^{2}=r_{}^{2}\times \left(1-\sqrt{r_{}^{2}-}{r^{\prime }}_{0}^{2}\right)$ $\mathrm{and}\;\mathrm{parameters}\;r^{2}\;\mathrm{and}\;r_{0}^{2}\;$are denoted as follows: $r_{0}^{2}=1-\frac{\sum^{\;}{\left(Y_{\mathrm{obs}}-k\times Y_{\mathrm{pred}}\right)}^{2}}{\sum^{\;}{\left(Y_{\mathrm{obs}}-{\bar{Y}}_{\mathrm{obs}}\right)}^{2}}$ and ${r^{\prime }}_{0}^{2}=1-\frac{\sum^{\;}{\left(Y_{\mathrm{pred}}-k^{\prime }\times Y_{\mathrm{obs}}\right)}^{2}}{\sum^{\;}{\left(Y_{\mathrm{pred}}-{\bar{Y}}_{\mathrm{pred}}\right)}^{2}}$ The terms k and k^′^ are explained as follows: $k=\frac{\sum^{\;}{\left(Y_{\mathrm{obs}}\times Y_{\mathrm{pred}}\right)}^{}}{\sum^{\;}{\left(Y_{\mathrm{pred}}\right)}^{2}}$$\mathrm{and}\;k^{\prime }=\frac{\sum^{\;}{\left(Y_{\mathrm{obs}}\times Y_{\mathrm{pred}}\right)}^{}}{\sum^{\;}{\left(Y_{\mathrm{obs}}\right)}^{2}}$	0.0173 and 0.9181	$\Delta r_{m}^{2}$$<\;0.2\;\mathrm{provided}\;\mathrm{that}\;\mathrm{the}\;\mathrm{value}\;\mathrm{of}\;\bar{r_{m}^{2}}$ 2 > 0.5	They reflect the overall predictability of the model for the entire data set.
$\mathrm{PRESS}=\sum^{\;}{\left(Y_{\mathrm{obs}}-Y_{\mathrm{pred}}\right)}^{2}$	0.3446		It evaluates the model using the predicted residual sum of squares.
$\mathrm{SDEP}=\sqrt{\frac{\mathrm{PRESS}}{n}}$	0.1252		Standard deviation of error of prediction (SDEP) is calculated from PRESS.
$\mathrm{MAE}=\frac{\sum^{\;}\left|Y_{\mathrm{obs}}-Y_{\mathrm{pred}}\right|}{n}$	0.0772		Index of errors in the context of predictive modeling studies.

**Table 6:**
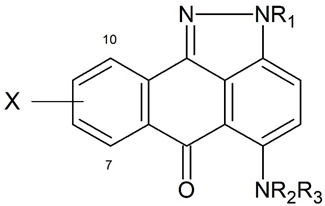
Chemical structures and antitumor activity of the anthrapyrazoles studied.

Compound	Set	X	R_1_	NR_2_R_3_	L1210 Leukemia In Vitro: IC_50_,M
a-01	training	H	H	NHCH_2_CH_2_NHCH_2_CH_2_OH	2.2 × 10^−6^
a-02	test	H	H	NHCH_2_CH_2_NEt_2_	1.5 × 10^−6^
a-03	test	H	CH_3_	NHCH_2_CH_2_NHCH_2_CH_2_OH	7.1 × 10^−7^
a-04	training	H	CH_3_	NHCH2CH_2_NEt_2_	6.7 × 10^−7^
a-07	training	H	CH_2_CH_2_OH	NHCH_2_CH_2_NHCH_2_CH_2_OH	1.8 × 10^−6^
a-08	training	H	CH_2_CH_2_OH	NHCH_2_CH_2_NEt_2_	8.8 × 10^−6^
a-14	test	H	CH_2_CH_2_NH_2_	NHCH_2_CH_2_NHCH_2_CH_2_OH	8.0 × 10^−8^
a-15	training	H	CH_2_CH_2_NHCH_2_CH_2_OH	NHCH_3_	7.4 × 10^−7^
a-16	test	H	CH_2_CH_2_NHCH_2_CH_2_OH	NHCH_2_CH_2_OH	7.5 × 10^−7^
a-17	training	H	CH_2_CH_2_NHCH_2_CH_2_OH	NHCH_2_CH_2_NH_2_	6.9 × 10^−8^
a-18	training	H	CH_2_CH_2_NHCH_2_OH	NHCH_2_CH_2_NHCH_2_CH_2_OH	7.4 × 10^−8^
a-19	training	H	CH_2_CH_2_NHCH_2_CH_2_OH	NHCH_2_CH_2_NMe_2_	3.2 × 10^−8^
a-20	training	H	CH_2_CH_2_NHCH_2_CH_2_OH	NHCH_2_CH_2_NEt_2_	6.0 × 10^−8^
a-21	training	H	CH_2_CH_2_NEt_2_	NH(CH_2_)_5_CH_3_	2.0 × 10^−6^
a-23	training	H	CH_2_CH_2_NEt_2_	NHCH_2_CH_2_NH_2_	4.6 × 10^−8^
a-24	training	H	CH_2_CH_2_NEt_2_	NHCH_2_CH_2_NHMe	2.7 × 10^−8^
a-25	training	H	CH_2_CH_2_NEt_2_	NHCH_2_CH_2_NHCH_2_CH_2_OH	3.2 × 10^−8^
a-26	test	H	CH_2_CH_2_NEt_2_	NHCH_2_CH_2_NEt_2_	3.9 × 10^−7^
a-27	training	H	CH_2_CH_2_NEt_2_	NH(CH_2_)_3_NEt_2_	5.2 × 10^−7^
a-28	training	H	CH_2_CH_2_NEt_2_	NH(CH_2_)_4_NEt_2_	6.2 × 10^−7^
a-29	training	H	CH_2_CH_2_NEt_2_	NH(CH_2_)_7_NEt_2_	6.3 × 10^−7^
a-30	test	H	CH_2_CH_2_NEt_2_	NHCH_2_CH_2_N(CH_2_CH_2_)_2_O	4.8 × 10^−7^
a-31	test	H	CH_2_CH_2_NEt_2_	NHCH2CH_2_N(CH_2_CH_2_)_2_NH	5.0 × 10^−7^
a-32	training	H	CH_2_CH_2_NEt_2_	NHCH2CH_2_N(CH_2_CH_2_)_2_NCOOCH_2_Ph	3.9 × 10^−7^
a-33	training	7,10-(OH)_2_	CH_3_	NHCH_2_CH_2_NH_2_	2.4 × 10^−7^
a-34	training	7,10-(OH)_2_	CH_3_	NHCH_2_CH_2_NHCH_2_CH_2_OH	1.5 × 10^−7^
a-35	training	7,10-(OH)_2_	CH_3_	NHCH_2_CH_2_NEt_2_	4.5 × 10^−7^
a-36	training	7,10-(OH)_2_	CH_2_Ph	NHCH_2_CH_2_NMe_2_	8.6 × 10^−7^
a-38	training	7,10-(OH)_2_	CH_2_CH_2_OMe	NHCH_2_CH_2_NHCH_2_CH_2_OH	1.6 × 10^−6^
a-40	training	7,10-(OH)_2_	CH_2_CH_2_OH	NHCH_2_CH_2_NH_2_	4.8 × 10^−7^
a-41	training	7,10-(OH)_2_	CH_2_CH_2_OH	NHCH_2_CH_2_NHCH_2_CH_2_OH	7.8 × 10^−7^
a-42	training	7,10-(OH)_2_	CH_2_CH_2_OH	NHCH_2_CH_2_NMe_2_	1.5 × 10^−8^
a-43	training	7,10-(OH)_2_	CH_2_CH_2_OH	NHCH_2_CH_2_NEt_2_	7.3 × 10^−7^
a-44	training	7,10-(OH)_2_	CH_2_CH_2_OH	NHCH_2_CH_2_N(CH_2_CH_2_)_2_O	1.1 × 10^−6^
a-46	training	7,10-(OH)_2_	CH_2_CH(OH)CH_2_OH	NHCH_2_CH_2_NHCH_2_CH_2_NMe_2_	2.2 × 10^−6^
a-47	test	7,10-(OH)_2_	CH_2_CH_2_NH_2_	NHCH_2_CH_2_NH_2_	4.8 × 10^−7^
a-48	training	7,10-(OH)_2_	CH_2_CH_2_NH_2_	NH(CH_2_)_3_NH_2_	3.1 × 10^−7^
a-49	test	7,10-(OH)_2_	CH_2_CH_2_NH_2_	NHCH_2_CH_2_NHMe	7.0 × 10^−7^
a-50	training	7,10-(OH)_2_	CH_2_CH_2_NH_2_	NHCH_2_CH_2_NHCH_2_CH_2_OH	5.8 × 10^−7^
a-51	test	7,10-(OH)_2_	CH_2_CH_2_NH_2_	NH(CH_2_)_3_NHCH_2_CH_2_OH	8.7 × 10^−7^
a-52	test	7,10-(OH)_2_	CH_2_CH_2_NH_2_	NHCH_2_CH_2_NHCH_2_CH_2_NMe_2_	9.3 × 10^−7^
a-53	test	7,10-(OH)_2_	(CH_2_)_3_NH_2_	NHCH_2_CH_2_NHCH_2_CH_2_OH	1.6 × 10^−7^
a-54	test	7,10-(OH)_2_	(CH_2_)_3_NH_2_	NHCH_2_CH_2_NHCH_2_CH_2_NMe_2_	6.4 × 10^−7^
a-55	training	7,10-(OH)_2_	CH_2_CH_2_NHMe	NHCH_2_CH_2_NHCH_2_CH_2_OH	4.4 × 10^−7^
a-56	training	7,10-(OH)_2_	CH_2_CH_2_NHCH_2_CH_2_OH	NHCH_2_CH_2_NH_2_	1.6 × 10^−6^
a-57	training	7,10-(OH)_2_	CH_2_CH_2_NHCH_2_CH_2_OH	NH(CH_2_)_3_NH_2_	9.6 × 10^−7^
a-60	training	7,10-(OH)_2_	CH_2_CH_2_NHCH_2_CH_2_OH	NHCH_2_CH_2_NHMe	1.4 × 10^−7^
a-62	training	7,10-(OH)_2_	CH_2_CH_2_NHCH_2_CH_2_OH	NHCH_2_CH_2_NHCH_2_CH_2_OH	7.4 × 10^−7^
a-63	training	7,10-(OH)_2_	CH_2_CH_2_NHCH_2_CH_2_OH	NH(CH_2_)_3_NHCH_2_CH_2_OH	1.8 × 10^−6^
a-64	test	7,10-(OH)_2_	CH_2_CH_2_NHCH_2_CH_2_OH	NHCH_2_CH_2_N(CH_2_CH_2_OH)_2_	4.3 × 10^−7^
a-65	training	7,10-(OH)_2_	CH_2_CH_2_NHCH_2_CH_2_OH	NH(CH_2_)_3_N(CH_2_CH_2_OH)_2_	9.2 × 10^−7^
a-66	training	7,10-(OH)_2_	CH_2_CH_2_NHCH_2_CH_2_OH	NHCH_2_CH_2_NMe_2_	2.3 × 10^−7^
a-67	training	7,10-(OH)_2_	CH_2_CH_2_NHCH_2_CH_2_OH	NHCH_2_CH_2_NEt_2_	5.1 × 10^−7^
a-68	training	7,10-(OH)_2_	CH_2_CH_2_NHCH_2_CH_2_OH	NHCH_2_CH_2_N(CH_2_CH_2_)_2_O	6.5 × 10^−7^
a-69	training	7,10-(OH)_2_	CH_2_CH_2_NHCH_2_CH_2_OH	N(CH_2_CH_2_)_2_NMe	4.3 × 10^−7^
a-70	test	7,10-(OH)_2_	CH_2_CH_2_NHCH_2_CH_2_OH	NHCH_2_CH_2_NHCH_2_CH_2_NH_2_	3.3 × 10^−7^
a-71	training	7,10-(OH)_2_	CH_2_CH_2_NHCH_2_CH_2_OH	NHCH_2_CH_2_NHCH_2_CH_2_NMe_2_	7.6 × 10^−7^
a-73	training	7,10-(OH)_2_	CH_2_CH_2_NHCH_2_CH_2_OH	N(Me)CH_2_CH_2_NMe_2_	6.3 × 10^−7^
a-74	training	7,10-(OH)_2_	CH_2_CH_2_NHCH_2_CH_2_OH	NH(CH_2_)_3_NH2	1.8 × 10^−6^
a-76	training	7,10-(OH)_2_	CH_2_CH_2_NMeCH_2_CH_2_OH	NHCH_2_CH_2_NHCH_2_CH_2_OH	3.3 × 10^−7^
a-77	training	7,10-(OH)_2_	CH_2_CH_2_NMe_2_	NHCH_2_CH_2_NH_2_	2.2 × 10^−7^
a-78	training	7,10-(OH)_2_	CH_2_CH_2_NMe_2_	NH(CH_2_)_3_NH_2_	5.4 × 10^−7^
a-79	test	7,10-(OH)_2_	CH_2_CH_2_NMe_2_	NHCH_2_CH_2_NHCH_2_CH_2_OH	1.2 × 10^−7^
a-80	test	7,10-(OH)_2_	(CH_2_)_3_NMe_2_	NHCH_2_CH_2_NH_2_	2.2 × 10^−6^
a-81	test	7,10-(OH)_2_	(CH_2_)_3_NMe_2_	NH(CH_2_)_3_NH_2_	8.0 × 10^−7^
a-82	test	7,10-(OH)_2_	(CH_2_)_3_NMe_2_	NHCH_2_CH_2_NHCH_2_CH_2_OH	5.9 × 10^−7^
a-83	training	7,10-(OH)_2_	CH_2_CH_2_NEt_2_	NHCH_2_CH_2_NH_2_	4.6 × 10^−8^
a-84	test	7,10-(OH)_2_	CH_2_CH_2_NEt_2_	NHCH_2_CH_2_NHMe	7.4 × 10^−6^
a-86	training	7,10-(OH)_2_	CH_2_CH_2_NEt_2_	NHCH_2_CH_2_NHCH_2_CH_2_OH	1.3 × 10^−7^
a-87	test	7,10-(OH)_2_	CH_2_CH_2_NEt_2_	NHCH_2_CH_2_NEt_2_	5.5 × 10^−7^
a-88	test	7,10-(OH)_2_	CH_2_CH_2_NHCH_2_CH_2_NMe_2_	NHCH_2_CH_2_NHCH_2_CH_2_OH	1.4 × 10^−6^
a-90	training	7,10-(OH)_2_	CH_2_CH(OH)CH_2_NEt_2_	NHCH_2_CH_2_NHCH_2_CH_2_OH	8.4 × 10^−7^
a-91	training	7,10-(OH)_2_	CH_2_CH(OH)CH_2_NEt_2_	NHCH_2_CH_2_NEt_2_	1.3 × 10^−6^

**Table 7:** Specification of MARSplines analysis.

Options	Values
Maximum number of basis functions	40
Degree of interactions	3
Penalty	2
Threshold	0.0005
Apply pruning	YES

## Supplementary Materials

The following supporting information can be downloaded at: https://www.mdpi.com/article/10.3390/ijms23095132/s1.
