# Supplementary material for: Application of Multivariate Adaptive Regression Splines (MARSplines) for Predicting Antitumor Activity of Anthrapyrazole Derivatives
Source: Int J Mol Sci. 2022 May 4;23(9):5132. doi: 10.3390/ijms23095132 (PMC9104800; doi:10.3390/ijms23095132)
Supplement: Supplementary file 1 [file ijms-23-05132-s001.zip › ijms-1641655-supplementary.pdf]

Table S1. Values of experimental and predicted data of antitumor activity of anthrapyrazoles studied.

Compound	Set	$pIC_{50\ exp}$	$pIC_{50\ calc}$	Δ
a-01	training	5.65757732	5.61923018	0.03834714
a-02	test	5.82390874	5.92584593	-0.10193719
a-03	test	6.14874165	6.12867153	0.02007012
a-04	training	6.17392520	6.26342706	-0.08950186
a-07	training	5.74472749	5.73348332	0.01124417
a-08	training	6.05551733	6.03384424	0.02167309
a-14	test	7.09691001	7.02934169	0.06756832
a-15	training	6.13076828	6.11602433	0.01474395
a-16	test	6.12493874	6.16825123	-0.04331249
a-17	training	7.16115091	6.95374675	0.20740416
a-18	training	7.13076828	7.13352053	-0.00275225
a-19	training	7.49485002	7.58204829	-0.08719827
a-20	training	7.22184875	7.08028247	0.14156628
a-21	training	5.69897000	5.84378942	-0.14481942
a-23	training	7.33724217	7.42750735	-0.09026518
a-24	training	7.56863624	7.49905287	0.06958337
a-25	training	7.49485002	7.49781744	-0.00296742
a-26	test	6.40893539	6.42809260	-0.01915721
a-27	training	6.28399666	6.39974011	-0.11574345
a-28	training	6.20760831	6.21571588	-0.00810757
a-29	training	6.20065945	6.16049104	0.04016841
a-30	test	6.31875876	6.36746770	-0.04870894
a-31	test	6.30103000	6.19624168	0.10478832
a-32	training	6.40893539	6.46575983	-0.05682444
a-33	training	6.61978876	6.58053033	0.03925843
a-34	training	6.82390874	6.85740690	-0.03349816
a-35	training	6.34678749	6.32453155	0.02225594
a-36	training	6.06550155	5.90923681	0.15626474
a-38	training	5.79588002	5.98801088	-0.19213086
a-40	training	6.31875876	6.28948074	0.02927802
a-41	training	6.10790540	6.16470031	-0.05679491
a-42	training	7.82390874	7.83348736	-0.00957862
a-43	training	6.13667714	6.31466476	-0.17798762
a-44	training	5.95860731	6.14435413	-0.18574682
a-46	training	5.65757732	5.64271170	0.01486562
a-47	test	6.31875876	6.42366972	-0.10491096
a-48	training	6.50863831	6.44412269	0.06451562
a-49	test	6.15490196	6.52542500	-0.37052304
a-50	training	6.23657201	6.22192657	0.01464544
a-51	test	6.06048075	6.10308113	-0.04260038
a-52	test	6.03151705	6.14501913	-0.11350208
a-53	test	6.79588002	6.56103082	0.23484920
a-54	test	6.19382003	6.20542398	-0.01160395
a-55	training	6.35654732	6.15840229	0.19814503
a-56	training	5.79588002	5.73407667	0.06180335
a-57	training	6.01772877	6.04698435	-0.02925558

a-60	training	6.85387196	6.95011544	-0.09624348
a-62	training	6.13076828	6.20181874	-0.07105046
a-63	training	5.74472749	5.70108400	0.04364349
a-64	test	6.36653154	6.36288473	0.00364681
a-65	training	6.03621217	6.16734853	-0.13113636
a-66	training	6.63827216	6.61508816	0.02318400
a-67	training	6.29242982	6.34395317	-0.05152335
a-68	training	6.18708664	6.07022871	0.11685793
a-69	training	6.36653154	6.30977205	0.05675949
a-70	test	6.48148606	6.27034674	0.21113932
a-71	training	6.11918641	6.11131309	0.00787332
a-73	training	6.20065945	6.12736279	0.07329666
a-74	training	5.74472749	5.69953786	0.04518963
a-76	training	6.48148606	6.39179691	0.08968915
a-77	training	6.65757732	6.72268102	-0.06510370
a-78	training	6.26760624	6.35376268	-0.08615644
a-79	test	6.92081875	6.73720013	0.18361862
a-80	test	6.69897000	6.73603740	-0.03706740
a-81	test	6.09691001	6.15268527	-0.05577526
a-82	test	6.22914799	6.18301098	0.04613701
a-83	training	7.33724217	7.36613703	-0.02889486
a-84	test	5.13076828	5.09367634	0.03709194
a-86	training	6.88605665	6.75542806	0.13062859
a-87	test	6.25963731	6.18253845	0.07709886
a-88	test	5.85387196	5.91001908	-0.05614712
a-90	training	6.07572071	6.03175652	0.04396419
a-91	training	5.88605665	5.83038730	0.05566935

Table S2. Chemical structures of external set of anthrapyrazoles and antitumor activity against murine leukemia L1210 predicted by the optimal MARS model.

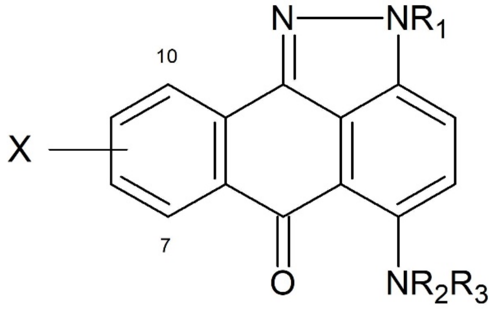

Compound	Set	X	R_1	NR_2R_3	Predicted L1210 Leukemia: pIC_{50},M
pd112,600	external	7,10-$(OH)_2$	$CH_2CH(OH)CH_2OH$	$NHCH_2CH_2NHCH_2CH_2OH$	5.24912034
pd112,145	external	7,10-$(OH)_2$	$CH_2CH_2NHCH_2CH_2N(CH_3)_2$	$NHCH_2CH_2NHCH_2CH_2OH$	6.07466913
pd113,785	external	7-OH	$CH_2CH_2NHCH_2CH_2OH$	$NHCH_2CH_2NHCH_2CH_2OH$	8.44756776
pd114,254	external	7,8,10-$(OH)_3$	$CH_2CH_2NHCH_2CH_2OH$	$NHCH_2CH_2NHCH_2CH_2OH$	5.68049669
pd114,817	external	7-OH	$CH_2CH_2NHCH_2CH_2OH$	$NHCH_2CH_2NHCH_3$	8.29128247
pd115,593	external	7,8,10-$(OH)_3$	$CH_2CH_2NHCH_2CH_2OH$	$NHCH_2CH_2NHCH_3$	6.60859232
pd116,522	external	7,10-$(OH)_2$	$CH_2CH_2NHCH_2CH_2OH$	NO_2	8.35698440
